# Epidemiological Study of *Betacoronaviruses* in Captive Malayan Pangolins

**DOI:** 10.3389/fmicb.2021.657439

**Published:** 2021-03-03

**Authors:** Linmiao Li, Xiaohu Wang, Yan Hua, Ping Liu, Jiabin Zhou, Jing Chen, Fuyu An, Fanghui Hou, Wenzhong Huang, Jinping Chen

**Affiliations:** ^1^Guangdong Key Laboratory of Animal Conservation and Resource Utilization, Guangdong Public Laboratory of Wild Animal Conservation and Utilization, Institute of Zoology, Guangdong Academy of Sciences, Guangzhou, China; ^2^Institute of Animal Health, Guangdong Academy of Agricultural Sciences, Guangzhou, China; ^3^Guangdong Provincial Key Laboratory of Silviculture, Protection and Utilization, Guangdong Academy of Forestry, Guangzhou, China; ^4^Guangdong Provincial Wildlife Rescue Center, Guangzhou, China

**Keywords:** *Betacoronavirus*, epidemiology, Malayan pangolin, ACE2, phylogenetic analysis

## Abstract

The coronavirus disease 2019 (COVID-19) outbreak has significantly affected international public health safety. It has been reported that the pathogen severe acute respiratory syndrome coronavirus 2 (SARS-CoV-2), which causes COVID-19, could originate from bats and utilize the Malayan pangolin (*Manis javanica*) as an intermediate host. To gain further insights into the coronaviruses carried by pangolins, we investigated the occurrence of *Betacoronavirus* (β-CoV) infections in captive Malayan pangolins in the Guangdong province of China. We detected three β-CoV-positive *M. javanica* individuals with a positive rate of 6.98% and also detected β-CoV in two dead pangolins sampled in August 2019. The CoV carried by pangolins is a new β-CoV, which is genetically related to SARS-CoV-2. Furthermore, the expression of angiotensin-converting enzyme 2 (ACE2) was detected in eight organs of pangolins, with the highest ACE2 mRNA levels in the kidney, suggesting that these organs could be at a risk of β-CoV infection. These results enable us to better understand the status of β-CoV carried by Malayan pangolins, while providing a theoretical basis for better pangolin protection and viral control.

## Introduction

The emergence of severe acute respiratory syndrome coronavirus 2 (SARS-CoV-2; provisionally named 2019 novel coronavirus or 2019-nCoV) has become a global challenge. The disease caused by SARS-CoV-2 was designated coronavirus disease 2019 (COVID-19) by the World Health Organization (WHO) (Wu et al., 2020; Zhou F. et al., 2020; Zhu et al., 2020) and was defined as a public health emergency of international concern on January 30, 2020. As of January 18, 2021, data from the WHO have confirmed 93,194,922 cases of COVID-19, including 2,014,729 deaths, indicating that this is a major global threat to public health. Based on whole genome sequence comparison, Bat-CoV-RaTG13 from the bat, *Rhinolophus affinis*, colonized in China’s Yunnan province, was found to be 96% identical to SARS-CoV-2, suggesting that SARS-CoV-2 could be of bat origin (Zhou P. et al., 2020). However, direct contact between bats and humans is rare as discovered in the SARS-CoV and MERS-CoV epidemics (Drosten et al., 2003; Guan et al., 2003; Zaki et al., 2012; Cunha and Opal, 2014; Drosten et al., 2014; Sabir et al., 2016); therefore, it seems more likely that the virus transmission route involves a spillover of SARS-CoV-2 to humans through an intermediate host rather than direct transmission from bats.

The existence of an intermediate animal host of SARS-CoV-2 between the bat reservoir and humans is currently under investigation. Researchers discovered a *Betacoronavirus* (β-CoV) closely related to the newly emerged SARS-CoV-2 in a metagenomic dataset from sampled pangolins (Lam et al., 2020; Liu et al., 2020; Xiao et al., 2020; Zhang et al., 2020). Furthermore, researchers believe that pangolins have the potential to act as an intermediate host of SARS-CoV-2, possibly from the recombination of a pangolin-CoV virus with a Bat-CoV-RaTG13 virus (Xiao et al., 2020). However, whole genome sequence comparison revealed that pangolin and human viruses share 90.3% of their whole genome and that the cleavage site between S1 and S2 in SARS-CoV-2 had multiple insertions (i.e., PRRA) when compared with those of Bat-CoV-RaTG13 and pangolin-CoV-2020 (Lam et al., 2020; Liu et al., 2020). Conversely, other researchers believe that pangolins may be a natural reservoir of SARS-CoV-2-like CoVs (Liu et al., 2020; Zhang et al., 2020); however, there has been no evidence of coronavirus or other potentially zoonotic viral emergence from the Malayan pangolins (*Manis javanica*) entering the wildlife trade through Malaysia (Lee et al., 2020). Nevertheless, pangolins are currently receiving extensive attention and debate over their role as potential hosts of SARS-CoV-2.

Angiotensin-converting enzyme 2 (ACE2) is a metalloprotease that is widely found in the heart, liver, lung, kidney, gastrointestinal tract, and other major animal organs (Crackower et al., 2002; Harmer et al., 2002). It plays a key role in regulating the heart and kidney functions and controlling blood pressure (Ingelfinger, 2009). In a previous study on SARS-CoV and SARS-CoV-2, ACE2 was found to be the main functional receptor for the two viruses. It is likely that pangolin-CoV uses ACE2 as its receptor because pangolin-CoV-2020 has a conserved receptor-binding domain (Liu et al., 2020). Thus, it is important to understand which pangolin tissues are susceptible to β-CoV using ACE2 expression analysis.

Currently, the world is facing an outbreak of COVID-19. Controlling the COVID-19 pandemic and tracing the virus and its transmission are the key tasks faced by humanity. Although pangolins have attracted attention as potential hosts of SARS-CoV-2, and recent studies have reported β-CoV in confiscated diseased or dead pangolins (Liu et al., 2019; Lam et al., 2020), β-CoV prevalence has not yet been reported in living and healthy pangolins. Therefore, an epidemiological investigation of β-CoVs in pangolins is needed. With this purpose in mind, the objectives of this study were to: (i) determine whether there is a relationship between pangolins and SARS-CoV-2 infections, and (ii) collect real-time data to help decision-making in the prevention and control of the pandemic.

## Materials and Methods

### Pangolin Sample Collection and Preparation

In February and March of 2020, a total of 43 captive pangolin individuals (41 Malayan pangolins and 2 Chinese pangolins) from the Wildlife Rescue Center of Dongguan, Shenzhen, and Shaoguan were investigated and sampled, as shown in Supplementary Table S1. We used the non-destructive sampling to collect throat swab, anal swab, urine, feces, and environmental samples.

Seven pangolin individuals (Supplementary Table S1), which were intercepted by Guangdong customs in July 2019, and later died, were sampled in August 2019. We collected 16 samples including 7 lungs, 5 lymph nodes, and 4 intestinal contents. Six of seven dead pangolins were tested for β-CoVs by viral metagenomics (Liu et al., 2020). The 16 samples were tested for β-CoVs by quantitative real-time polymerase chain reaction (qRT-PCR). Among the seven dead pangolins, three Malayan pangolin individuals were dissected in a sterile environment; samples, including the heart, liver, spleen, lung, kidney, stomach, lymph node, and pancreas, were used for qRT-PCR to determine the expression of ACE2 mRNA.

The samples were immediately placed in 2 mL polyethylene tubes (RNase-free) and stored in a liquid nitrogen tank. All samples were stored at −80°C until nucleic acid extraction. All lab personnel were professionally trained and wore protective clothing to protect against biological agents before sample collection.

### RNA Extraction

Viral nucleic acid was extracted using a QIAamp^®^ Viral RNA Mini Kit (Qiagen, Germany) after 112 samples were inactivated at 56°C for 30 min. A total of 50 mg of each sample (24 tissue samples from three dead pangolins) were ground into powder and transferred to diethylpyrocarbonate-treated Eppendorf tubes before being volatilized using liquid nitrogen. Total RNA was extracted using 1 mL RNAsio Plus (Takara, Japan), and 1 μg cDNA was synthesized using the SuperScript III First-Strand Synthesis System (Invitrogen, United States).

### Quantitative Real-Time Polymerase Chain Reaction (qRT-PCR)

First, all samples were detected using the 2019-nCoV Nucleic Acid Test Kit (dual fluorescence method; Genekey, Shenzhen, China), which was designed based on the ORF1ab and N gene fragments of the SARS-CoV-2 genome. The selection criteria were as follows: Ct ≤ 35, positive; Ct < 40, negative; if 35 < Ct < 40, then the samples needed to be tested once again and were considered positive when the Ct value was < 40; otherwise, they were considered negative. Second, all samples were detected again using qRT-PCR using the primers and probe (N-F, 5′-GGGGAACTTCTCCTGCTAGAAT-3′; N-R, 5′-CAGACATTTTGCTCTCAAGCTG-3′; and N-P, 5′-FAM-TTGCTGCTGCTTGACAGATT-TAMRA-3′) based on the N gene of new coronavirus nucleic acids as announced by the Chinese Center for Disease Control and Prevention of Viral Diseases. The Centers for Disease Control (CDC)-positive plasmid combining the N gene sequence was synthesized at Sangon Biotech (Shanghai) Co., Ltd. by referring to the region of the viral genome. A 25 μL reaction was set up containing 5 μL of RNA, 12.5 μL of 2x reaction buffer, provided with the Superscript III one step RT-PCR system with Platinum Taq Polymerase (containing 0.4 mM of each deoxyribonucleotide triphosphates (dNTP) and 3.2 mM magnesium sulfate; Invitrogen, United States), 1 μL of reverse transcriptase/Taq mixture from the kit, 0.4 μL of a 50 mM magnesium sulfate solution (Invitrogen, United States), 1 μL of non-acetylated bovine serum albumin (1 mg/mL) (Roche, Switzerland), 2.6 μl H_2_O (RNase-free), 1 μL forward primer (10 μM), 1 μL reverse primer (10 μM), and 0.5 μL of probe (10 μM). Thermal cycling was performed at 50°C for 2 min for reverse transcription, followed by 95°C for 2 min, and 40 cycles of 95°C for 15 s and 60°C for 30 s. The selection criteria were as follows: Ct < 37, positive; Ct > 40, negative; if samples were 37 < Ct < 40, then they needed to be tested once again to be considered positive when the Ct value was below 40; otherwise, they were considered negative. Finally, the results of the two experiments were combined to determine the positivity or negativity of SARS-CoV-2 in pangolins.

qRT-PCR was used to determine the mRNA expression of ACE2 in different tissues of Malayan pangolins. The total volume of the qRT-PCR mixture was 20 μL, which included 10 μL 2x qPCR mixture (Takara, Japan), 0.5 μL of forward and reverse primers, 1 μL of template, with the remaining volume made up of nuclease-free water. The following procedure was used for amplification: 95°C for 30 s, 40 cycles of 95°C for 5 s, 60°C for 34 s, and 95°C for 15 s. β-actin was used as the internal control. The primers for pangolins were: ACE2-F, 5′-GCGTATGAATGGAACGACAGT-3′; ACE2-R, 5′-GAATGACGGCAGACACATTTT-3′; β-actin-F, 5′-TGCCCATCTACGAAGGTTATG-3′; and β-actin-R, 5′-GCA CAGCTTCTCCTTGATGTC-3′.

The expression of ACE2 mRNA was separately determined using the comparative CT (2^–△△*CT*^) method. The mean and standard deviation (M ± SD) were calculated from biological replicates. Statistical significance was measured using the independent samples *t*-test by SPSS 17.0 software.

### RT-PCR and Sequencing

To confirm the positivity accuracy of the previously tested samples, we used RT-PCR with primers targeting against fragments from the N gene to the open reading frame (ORF)-10 gene of SARS-CoV-2 and pangolin-CoV. The specific primers were: P24F, 5′-AACTCAAGCCTTACCGCAGA-3′; P24R, 5′-ATAGCCCATCTGCCTTGTGT-3′; and size, 160 nucleotides (nt). A 25 μL reaction was set up, containing 12.5 μL PCR mix (Gentech, China), 10.5 μl double-distilled water (dd H_2_O), 1 μL template, 0.5 μL Primer P24F (10 μM), and 0.5 μL Primer P24R (10 μM). Thermal cycling was performed at 94°C for 3 min, followed by 35 cycles at 94°C for 30 s, 50°C for 30 s, and 72°C for 1 min, followed by 72°C for 1 min. We used the template from a pangolin lung (individual number: L08) as a positive control (Liu et al., 2019). PCR products were detected on 1.5% agarose gel and sequenced. These sequences were compared using MEGA X (Sudhir et al., 2018).

### Phylogenetic Analysis

We downloaded 26 fragment sequences of N gene from GenBank (Supplementary Table S2). Phylogenetic analyses were performed based on their nucleotides using IQ-Tree (Nguyen et al., 2015), and the evolutionary history was inferred by using the maximum likelihood method; the thread count and bootstrap values were 24 and 1,000, respectively.

## Results

### Field Investigation and Test Results of Pangolins

In February and March of 2020, we investigated the living conditions of pangolins in the Wildlife Rescue Centers of Dongguan, Shenzhen, and Shaoguan; all the pangolins were healthy and without pathological symptoms. Pangolin samples were randomly collected (Supplementary Table S1). The Ct values of three samples after two detections by qRT-PCR using the same primer were all less than 40, so three stool samples were positive; however, the Ct values of two samples after one detection using qRT-PCR were all less than 40, so two stool samples were suspected to be positive (Supplementary Table S3). Meanwhile, one lymph node sample was positive and one intestinal content sample was suspected to be positive in two dead pangolin individuals.

Finally, five of seven samples were identified as positive using RT-PCR and sequencing, including three feces (named DG13-3, DG14-3, DG18-3), one lymph node (named GZ1-2), and one intestinal content (named GZ5-2), from three healthy and two dead *M. javanica* individuals. The overall positivity rate was 10.00% for 50 pangolins individuals and 6.98% for the 43 living pangolin individuals.

### Sequence Analysis of Positive Samples

**FIGURE 1:**
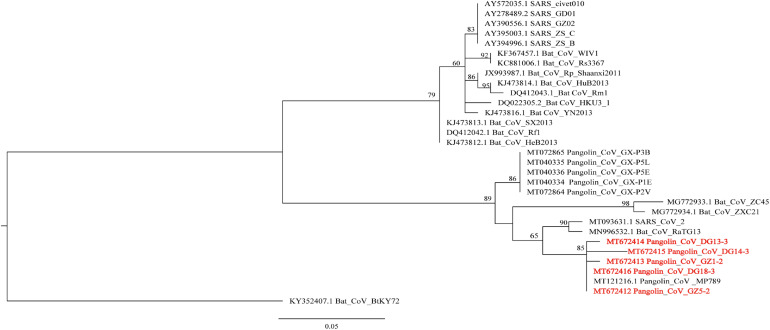
Phylogenetic relationship of N gene of β-CoV from Malayan pangolin and other hosts. The analysis was inferred using the Maximum Likelihood method based on IQ-Tree (Nguyen et al., 2015). Branch bootstrap values are shown and were based on 1,000 replicates. The red letters indicate sequences of the β-CoV from *M. javanica* in this study.

**FIGURE 2:**
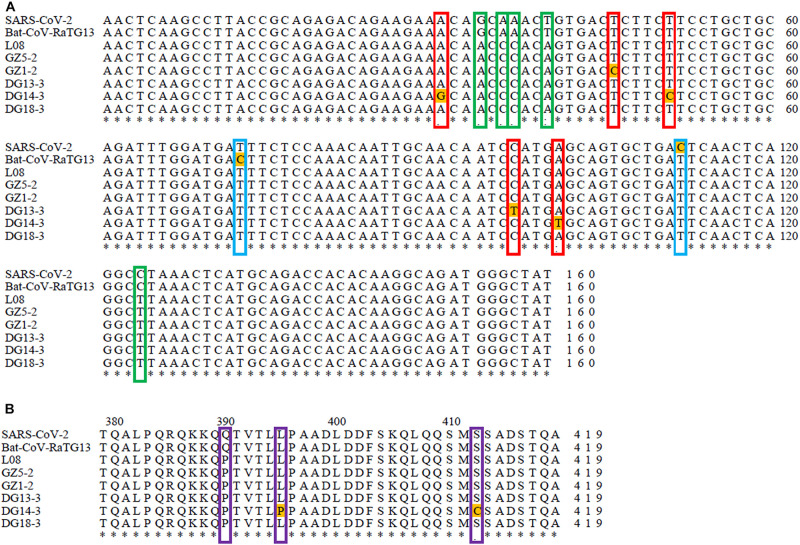
Sequence comparison between SARS-CoV-2, Bat-CoV-RaTG13, and pangolin-CoVs based on their N gene sequence: **(A)** Sequence comparison between SARS-CoV-2, Bat-CoV-RaTG13, and pangolin-CoVs based on their N gene nucleotide sequence. The red frame indicates nucleotide variation sites among pangolins. The green frame indicates nucleotide variation sites between pangolins and SARS-CoV-2 or Bat-RaTG13. The blue frame indicates nucleotide variation sites between SARS-CoV-2 and Bat-RaTG13. **(B)** Sequence comparison between SARS-CoV-2, Bat-CoV-RaTG13, and pangolin-CoVs based on their N gene amino acid sequence. The purple frame indicates amino acid variation sites between pangolins and SARS-CoV-2 or Bat-RaTG13. The asterisk indicates that all nucleotide and amino acid sites are exactly the same in all compared sequences.

The N protein is a highly conserved and immunogenic phosphoprotein present in CoVs. Therefore, the CoV N protein has often been used as a marker in diagnostic assays. Phylogenetic analysis showed that the relationship of the five sequences (GenBank number: MT672412-MT672416) from positive samples were closer to the pangolin-CoV-MP789 carried by pangolin from Guangdong (Liu et al., 2020) than SARS-CoV-2 (Figure 1). The nucleotide sequence identities were 98.13–100% between five positive sequences and pangolin-CoV-MP789, as well as 93.75–95.63% between five positive sequences and SARS-CoV-2, respectively (Table 1). Therefore, the CoV carried by pangolins is a new β-CoV with variation in nucleotides and amino acids among pangolin individuals (Figures 2A,B).

**TABLE 1:** Nucleotide sequence identity of SARS-CoV-2, Bat-CoV-RaTG13 and pangolin-CoVs in 160 nucelotides (nt) from N gene sequence.

**Identity (%)**	**GZ5-2**	**GZ1-2**	**DG13-3**	**DG14-3**	**DG18-3**
SARS-CoV-2	95.63	95	95	93.75	95.63
Bat-CoV-RaTG13	95.63	95	95	93.75	95.63
Pangolin-CoV-MP789	100	99.38	99.38	98.13	100

### mRNA Expression of ACE2 Gene

**FIGURE 3:**
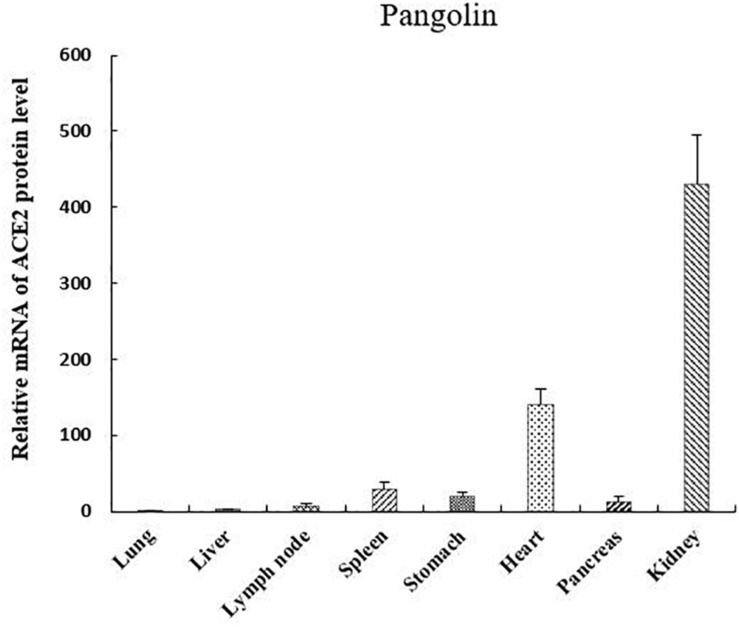
mRNA expression of angiotensin-converting enzyme 2 in different pangolin organs.

We investigated the mRNA expression levels of ACE2 in eight pangolin organs using qRT-PCR. We found that the mRNA expression levels of ACE2 were highest in the kidney; moreover, its expression levels were also high in the heart and spleen (Figure 3). The relative mRNA expression levels in tissues relevant to SARS-CoV-2 infection, such as the lungs were 400-fold lower than those in the kidneys. Among these organs, pangolins exhibited the lowest mRNA levels in the lung, followed by the pancreas, lymph, and liver. Our results suggest that all these organs are at risk of infection by SARS-CoV-2 or pangolin-CoV.

## Discussion

Traceability studies of SARS-CoV-2 revealed that pangolins might be an intermediate host for SARS-CoV-2 (Lam et al., 2020; Xiao et al., 2020); however, there is no strong evidence currently available to support this notion. Although studies have suggested that bats could be natural hosts for SARS-CoV-2 (Zhou P. et al., 2020), how the virus accomplished cross-species transfer and propagation remainunclear. Therefore, it is important to investigate whether living pangolins carry SARS-CoV-2. The results of the 43 pangolins from Dongguan, Shenzhen, and Shaoguan revealed that the positive rate of SARS-CoV-2-like virus was 6.98% in living individuals. We also detected β-CoV using RT-PCR in two dead pangolins from Guangzhou and one was also positive by metagenomic sequencing (Liu et al., 2020). Our research, to the best of our knowledge, is the first comprehensive study on captive pangolins carrying a new coronavirus similar to SARS-CoV-2 in Guangdong province. We detected coronavirus cases in healthy, symptomatic, and dead Malayan pangolins. This suggests that pangolins can carry and spread the virus; however, we cannot be certain if Malayan pangolins are natural coronavirus hosts (Tang et al., 2020). Therefore, it is necessary to strengthen worldwide cooperation to detect coronaviruses in wild populations of Malay pangolins. Recent research has revealed that the Malayan pangolin from both Peninsular Malaysia and Sabah did not carry coronavirus or other zoonotic viruses before entering the wildlife trade. This may be attributed to the fact that they were yet to be exposed to multiple potential sources of infection (Lee et al., 2020). Therefore, it is still necessary to detect coronaviruses in pangolins from their native countries to confirm whether pangolins are a natural host of the pangolin-CoV.

Coronavirus is mainly present in the respiratory and gastrointestinal systems of mammals and birds (Masters, 2006). Scientists have discovered that feces may be an important mode of transmission for some new coronaviruses carried by bats (Valitutto et al., 2020). Similarly, we found that coronavirus was mainly present in pangolin feces, indicating that this virus may also be transmitted through feces. Although pangolins in Dongguan live together, only 3 of 22 feces were found to be positive, and their sequences had nucleic acid variations with two different genotypes. This finding indicated that this coronavirus had a limited ability to spread among individuals. Although it was weakly infectious, it is important to prevent the pangolin virus from spreading to humans through fecal transmission. Interestingly we also identified coronavirus in the lymph nodes of dead pangolins, suggesting that this virus could attack the immune system. It is likely that pangolin-CoV uses ACE2 as its receptor (Liu et al., 2019; Xiao et al., 2020). ACE2 expression has been observed in multiple pangolin organs, including the heart, liver, spleen, lungs, kidneys, stomach, lymph nodes, and pancreas, implying that all of these organs are at risk of infection. Similarly, ACE2 expression has also been observed in the human kidneys, respiratory tract, lungs, ileum, bladder, esophagus, and heart. Moreover, SARS-CoV-2 directly infects the kidneys, causing acute renal failure with severe acute tubular necrosis and lymphocyte infiltration (Diao et al., 2020; Wadman et al., 2020). During examination of the dead pangolin named M1 (Supplementary Table S1), hemorrhage of the heart, liver, and kidney was observed, which could be related to coronavirus infection.

Our results suggest that pangolins may have been infected in markets or during trade-related activities, while being kept in overcrowded conditions with other animal species. Therefore, to further determine the source of pangolin-CoV, it is necessary to sample more pangolins, and to also examine other animals that live in close proximity in markets or during trade-related activities. Although we detected SARS-CoV-2 like viruses in some captive pangolins, the role of pangolins in the transmission of SARS-CoV-2 from a putative natural bat host to humans is yet to be established. Recently, wild animals from Southeast Asia have been smuggled into China (Heinrich et al., 2017), and little is known about the evolutionary status of the virus in these wild animals. However, sporadic coronavirus infections in both Guangdong and Guangxi in recent years indicate that we cannot ignore these potential hosts (Dong et al., 2007; Liu et al., 2019; Lam et al., 2020). Therefore, it is important to monitor and understand the coronavirus spectrum carried by animals over an extended period, (especially pangolins, confiscated in China) and to catalog the diversity of these viruses in their natural hosts, so that in the future we can more easily identify the host species if another coronavirus crosses into humans. This will be of great significance in protecting the health of pangolins and humans. In order to facilitate further in-depth research, collaboration is needed across multiple countries and institutions.

## Data Availability Statement

The datasets presented in this study can be found in online repositories. The names of the repository/repositories and accession number(s) can be found below: https://www.ncbi.nlm.nih.gov/genbank/, MT672412; https://www.ncbi.nlm.nih.gov/genbank/, MT672413; https://www.ncbi.nlm.nih.gov/genbank/, MT672414; https://www.ncbi.nlm.nih.gov/genbank/, MT672415; and https://www.ncbi.nlm.nih.gov/genbank/, MT672416.

## Ethics Statement

The animal study was reviewed and approved by the Committee on the Ethics of Animal Experiments of the Institute of Zoology of Guangdong Academy of Sciences.

## Author Contributions

JPC conceived the study. YH, JZ, FA, FH, and WH collected the samples. LL, XW, JZ, and JC performed virus detection and sequencing. LL, XW, and JPC performed the analysis. LL and JPC wrote and revised the manuscript. All authors contributed to the article and approved the submitted version.

## Conflict of Interest

The authors declare that the research was conducted in the absence of any commercial or financial relationships that could be construed as a potential conflict of interest.

## Supplementary Material

Supplementary Table 1 Sample information of pangolins.

Supplementary Table 2 Accession numbers and strain IDs of coronavirus strains isolated from different hosts.

Supplementary Table 3 Results of viral detection using quantitative real-time polymerase chain reaction.
